# Comparison of Shoulder Kinematics and Muscle Activation of Female Elite Handball Players With and Without Pain—An Explorative Cross-Sectional Study

**DOI:** 10.3389/fspor.2022.868263

**Published:** 2022-05-24

**Authors:** Tina Piil Torabi, Birgit Juul-Kristensen, Mogens Dam, Mette K. Zebis, Roland van den Tillaar, Jesper Bencke

**Affiliations:** ^1^Department of Sports Science, Nord University, Levanger, Norway; ^2^Human Movement Analysis Laboratory, Department of Orthopaedic Surgery, Copenhagen University Hospital, Hvidovre, Denmark; ^3^Department of Sports Science and Clinical Biomechanics, University of Southern Denmark, Odense, Denmark; ^4^Bülowsvej Physiotheraphy and Training, Frederiksberg, Denmark; ^5^Department of Midwifery, Physiotherapy, Occupational Therapy and Psychomotor Therapy, Faculty of Health, University College Copenhagen, Copenhagen, Denmark; ^6^Department of Orthopedic Surgery, Institute of Sports Medicine Copenhagen, Copenhagen University Hospital–Bispebjerg and Frederiksberg, Copenhagen, Denmark

**Keywords:** throwing kinematics, team handball, overhead athletes, EMG, prevention, shoulder pain, risk factors

## Abstract

Non-traumatic shoulder injuries are common in team handball. However, many athletes continue to throw, despite pain in the shoulder. This study investigated upper body kinematics and muscle activation while throwing in female elite handball players with and without shoulder pain. Thirty female elite team handball players, 15 with pain (age 22.2 ± 2.9 yrs.) and 15 without pain (age 20.4 ± 2.6 yrs.) performed five standing throws in which joint kinematics and muscle activity were measured in the following muscles: pectoralis major, infraspinatus, serratus anterior, latissimus dorsi, and upper-, middle-, and lower trapezius. The main findings revealed that peak joint angles and angular velocities were not different between groups; however, group differences were observed in earlier timing of position and longer time spent in maximal shoulder extension and external shoulder rotation in the pain group compared with the no pain group. The pain group also revealed a significant lower muscle peak activity in the serratus anterior during the cocking phase compared to the no pain group. After the cocking phase and at ball release, the groups had similar activation. In conclusion, the present study showed group differences in appearance and time spent in maximal humerus extension and external rotation and a different serratus anterior muscle peak activity between elite handball players playing with and without shoulder pain, which are identified as possible mechanisms of adaptation to avoid pain.

## Introduction

Handball is a popular worldwide sport. The International Handball Federation includes 180 countries and handball is part of the Olympic programme (Almeida et al., 2013). Handball is known for its complexity, e.g., with a high level of physical demands and variations combined with multiple technical skills (Chelly et al., 2011; Karcher and Buchheit, 2014; Michalsik et al., 2014, 2015; Michalsik and Aagaard, 2015). The impacts of physical contact between defenders and attackers have been estimated to appear every 22–36 s during a match situation (Michalsik et al., 2015). Throws are performed in different types with or without a jump and with different positions of the arm during throwing, depending on the position of the field and the activity of the defenders. The number of throws varies from playing position and during a match, and an average number of maximal throws has been registered to 4–15 at goal and 18–100 passing throws. The number of throws during training is believed to be significantly higher (Buchheit et al., 2009; Chelly et al., 2011; Karcher and Buchheit, 2014; Michalsik et al., 2014, 2015; Michalsik and Aagaard, 2015).

An overhead throw is categorized as an explosive movement that involves a full body kinetic chain and creates a high load at the shoulder. In handball, 44–75% of all athletes have a history of shoulder pain (Moller et al., 2012, 2017; Myklebust et al., 2013; Asker et al., 2018). Several studies have reported incidents of shoulder injuries among elite handball players between 9 and 58% (Moller et al., 2012; Myklebust et al., 2013; Asker et al., 2018). Shoulder pain has also been reported to have an impact on the athletes' performance in general and daily life activities (Mohseni-Bandpei et al., 2012; Clarsen et al., 2014). It is reported that regular handball regimes may cause overload shoulder injuries and that players often continue playing handball despite experiencing pain (Mohseni-Bandpei et al., 2012; Myklebust et al., 2013; Clarsen et al., 2014).

Several studies among overhead athletes have investigated risk factors for the appearance of shoulder pain, with a particular focus on physical and anatomic variations, where decreased glenohumeral (GH) range of motion (ROM), shoulder muscle strength (Trakis et al., 2008; Byram et al., 2010; Edouard et al., 2013; Tyler et al., 2014) and scapula control (Kibler et al., 1996, 2013a,b; Laudner et al., 2006, 2013; Myers et al., 2013; Pluim, 2013; Struyf et al., 2014) have been identified as risk factors. Abnormalities in these risk factors and increased training load are considered to increase the potential risk for shoulder pain in overhead athletes (Borsa et al., 2006; Moller et al., 2017).

None of the studies mentioned have investigated the kinematics of throwing alone, as a possible risk factor for shoulder injury, where a non-optimal throwing technique is anticipated to increase stress on the structures in the shoulder. An overhead throw is an open kinetic chain, where explosive power is generated from lower extremities, transferred trough the trunk and scapula to the end result of maximal ball velocity. The process requires the glenohumeral joint to balance between the two factors of mobility and stability within 2 s. A throw is divided into six phases, where the cocking phase, the point of maximal external shoulder rotation (MER) and the deceleration phase after ball release are considered the phases where most injuries occur (Borsa et al., 2006; Clarsen et al., 2014).

Several studies have performed kinematic and neuromuscular analyses on the three primary types of throws (standing, with run up and jump throws) performed in team handball (van den Tillaar and Ettema, 2009a,b, 2011; Wagner et al., 2010a,b, 2018; van den Tillaar and Cabri, 2012; Michalsik et al., 2015; Skejo et al., 2019). Furthermore, kinematic investigation shows how the main contributors to velocity in an overhead throw are the elbow extension and maximal shoulder internal rotation (Fradet et al., 2004; van den Tillaar and Ettema, 2007), and an increased MER during cocking phase (Stodden et al., 2005; van den Tillaar and Ettema, 2009a).

Prior studies have suggested different reasons for how pain occurs, but none have investigated the kinematics of throwing as a possible risk factor for shoulder injury, where a non-optimal or incorrect throwing technique is anticipated to increase stress on the structures in the shoulder (Borsa et al., 2006, 2008; Laudner et al., 2006; Myers et al., 2006, 2013; Trakis et al., 2008; Byram et al., 2010; Kibler et al., 2013b). A throw lasts for ~2 s and the muscular correct timing of the scapula-humeral muscles are important to generate optimal control of glenohumeral head. However, to our knowledge no neuromuscular investigation of the muscles considered to be important during an overhead throw has been performed in handball, nor have any previous study compared individual throwing technique with muscle activation patterns between healthy team handball players and those playing with shoulder pain.

This explorative study therefore investigated kinematics and muscle activation patterns during overhead standing handball throws to detect if any differences in throwing technique and/or neuromuscular activation patterns were present between healthy team handball players and those playing with shoulder pain.

## Materials and Methods

### Participants

**Figure 1:**
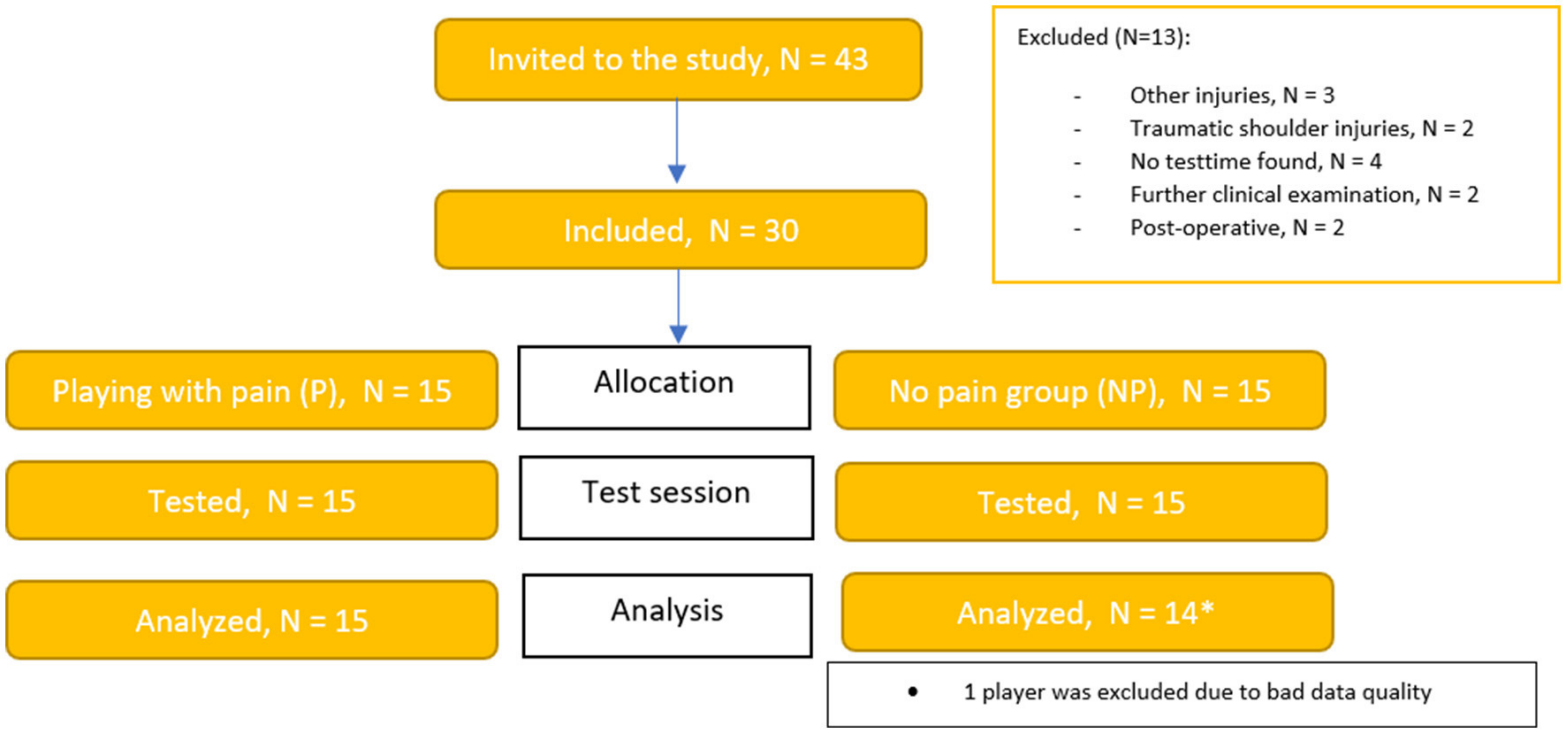
Flowchart with included subjects. The * symbol has a reference to the underlying white box, where the information of why one person has been eliminated from the analyze.

Forty-three female team handball players were invited to participate in the study, but only 30 were included. For this explorative study, 30 participants were considered sufficient as other similar studies investigating kinematics of throwing in handball have used the same or fewer subject (van den Tillaar and Cabri, 2012; Wagner et al., 2012, 2014). Thus, 15 players with shoulder pain (age 22.2 ± 2.9 yrs.) and 15 players without shoulder pain (age 20.4 ± 2.6 yrs.) were recruited from the top three best team handball leagues in Denmark and the best team handball league in Sweden (Figure 1).

### Inclusion and Exclusion Criteria

Coaches or staff of the medical team in 15 clubs received an invitation. Forty-three players replied to the questionnaire regarding pain, training and match exposure, and injury history. All participants included in the study were a minimum of 18 years old and right-handed. The participants had to play an active part in both the offensive and defensive part of the game and be part of handball training for a minimum of three times a week. Participants were excluded if they had missed a match within the last 6 weeks due to pain in the shoulder and/or if they reported pain, which was associated with a traumatic event or shoulder surgery.

The presence of shoulder pain was established by the Oslo Sports Trauma Research Center (OSTRC) questionnaire (Clarsen et al., 2013; Jorgensen et al., 2016). Further, custom-made questions were added to the protocol to record the type and amount of pain, and how it occurred. Finally, a telephone interview by a physical therapist was performed to determine whether the pain was traumatic or non-traumatic and to assess the history of shoulder pain. The participants included with pain where to report pain over the last 4 weeks. Participants included without pain should report no shoulder pain within the past 6 months prior to the invitation.

### Procedures

**Figure 2:**
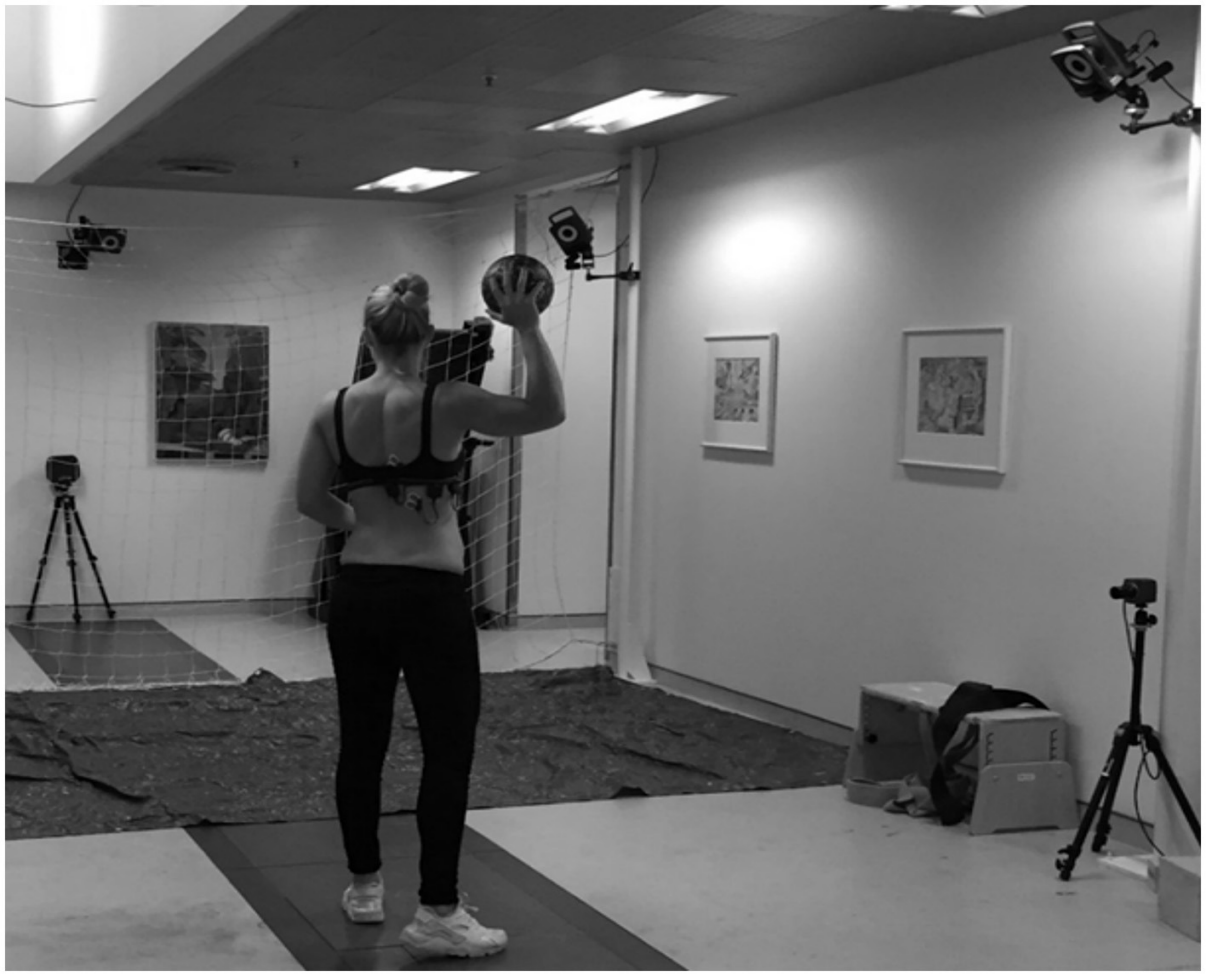
Measurement of shoulder kinematics and muscle activity during maximum throwing.

Participants did not play or exercise for a minimum of 24 h before testing. All participants performed a standardized warm-up protocol before the throwing test, which involved 20 standing trunk rotations, 10 × overhead squats, arm swings with both arms, walkout to plank, scapula push-ups, oscillated banded pull apart with an overhead reach and horizontal internal/external rotation and internal/external rotation with the arm placed beside truncus. After the warm-up protocol, participants performed 5–10 min of throwing/passing with the test instructor, until the participant felt ready to throw maximally. After the warm-up protocol, each participant performed five maximal standing throws (see Figure 2). The participants started standing with the contralateral leg in the front with the ball in both hands in front of the body. The participants were instructed to throw at maximal speed aiming at a square target area of 1 × 1 m. The test position was 7 m from a net with large meshes and participants were using a women's team handball (375 gr, diameter: 56 cm). A speedometer (Speedtrac X) was placed at the end of the room, behind the net, to collect data on throwing speed (Figure 2).

### Measurements

**Figure 3:**
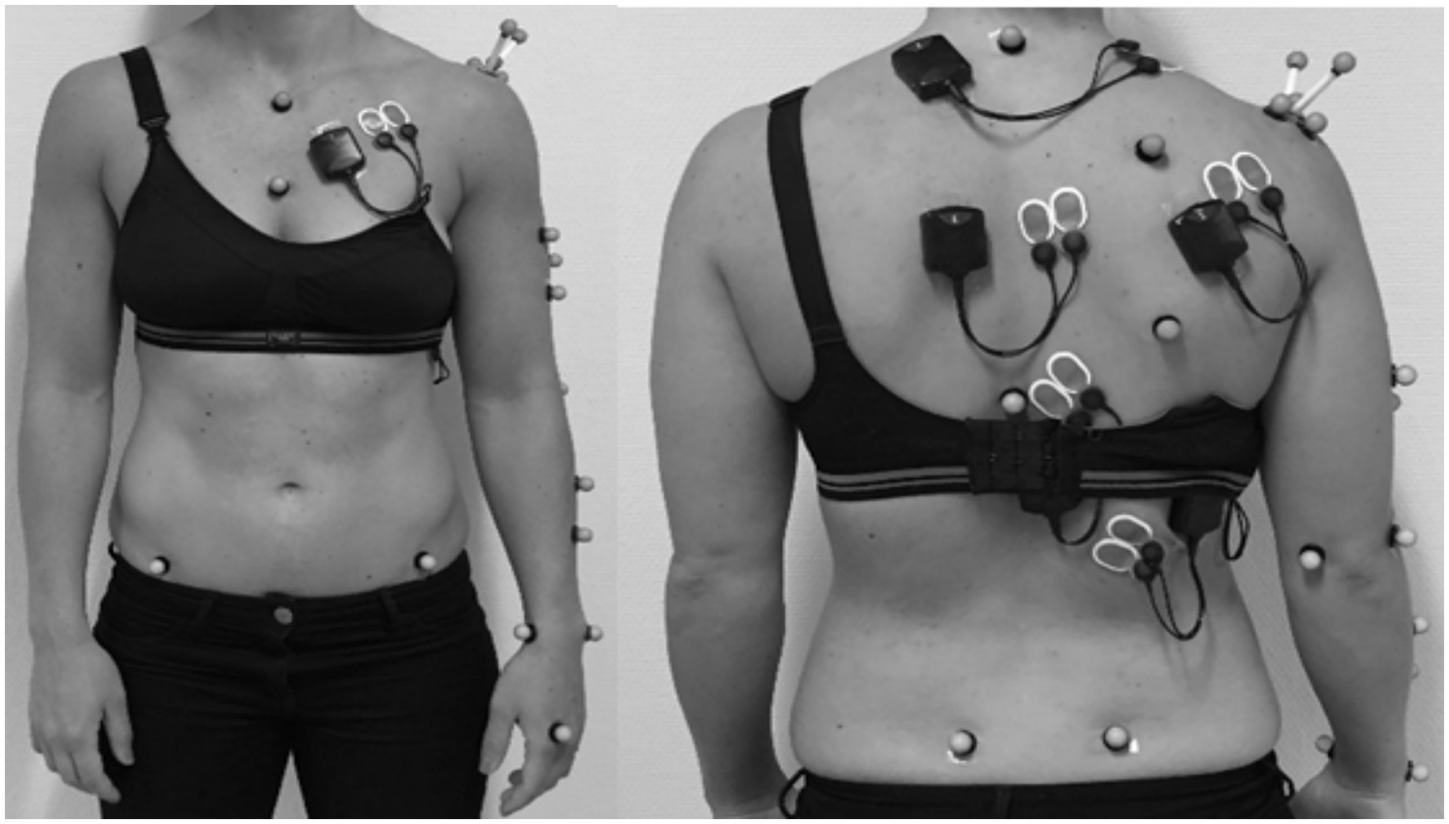
Position of markers for measuring shoulder kinematics and electrodes for measuring shoulder muscle activity.

The tests were performed in a biomechanics laboratory. Eight infrared Vicon T40 cameras (Vicon Motions Systems Ltd., Oxford, UK) were placed 2–6 m from the participant, and inherent software (Nexus 2.9, Vicon Motions Systems) was used to collect camera data and EMG data synchronously. A total of twenty-three 14-mm markers were placed over anatomical landmarks on the pelvis, thorax, scapula, upper and lower arm in accordance with the recommendations of the International Society of Biomechanics (Wu et al., 2005) (Figure 3).

Marker positions were obtained with a camera frequency of 200 Hz, and raw data were filtered using a Butterworth filter with a cut-off frequency of 20 Hz. The positions of the markers were used to define local coordinate systems (LCS) for each segment. A trial containing flexion-extension, abduction/adduction and circumduction served as a basis for calculation of the gleno-humeral joint center from the LCS of the scapula, defined by an acromion based cluster, and the,LCS of the upper arm defined by the upper arm markers (see Figure 2), using the SCoRE method, as described by Monnet et al. (2007). Based on the principles described by Wu and colleagues (Wu et al., 2005; Plummer and Oliver, 2017) joint angles were calculated using custom-made scripts in Matlab and Bodybuilder (Vicon Motion Systems Ltd) in 2 degrees-of -freedom for the wrist and elbow, and 3 degrees-of-freedom for the shoulder, trunk and pelvis.

Based on previous study by van den Tillaar and Cabri (2012) instant of ball release was defined as the time of maximal wrist flexion, and joint angles and muscle activation patterns are presented synchronized to this instant.

### Electromyography (EMG)

Prior to EMG placement, the skin of the participant was cleaned according to standard recommendations (Konrad, 2005). Bipolar surface EMG electrodes (Medicotest A-10-N, Ag/AgCl electrodes) were placed at 2-cm inter-electrode distance on the following muscles: upper, middle and lower trapezius, latissimus dorsi, infraspinatus, serratus anterior, and pectoralis major, according to standardized recommendations (Barbero et al., 2012). EMG signals were recorded by wireless transmitters (MYON Aktos, Prophysics SOL AB, Zürich, Schweiz). Raw EMG signals were sampled with a frequency of 1,000 Hz, pre-amplified and bandpass filtered (20–450 Hz). Subsequently, the EMG signals were high-pass filtered using a fourth-order Butterworth filter with a cut-off frequency of 10 Hz, rectified and subsequently smoothed by a fourth-order Butterworth low-pass filter with a cut-off frequency of 10 Hz. Maximal EMG activity was obtained during maximal isometric voluntary contraction (MVC) as previously recommended (Barbero et al., 2012), including three maximal isometric MVC's, each lasting 5 s with 30 s of rest between each contraction. The MVC trials were high-pass filtered, rectified, and lowpass filtered in the same manner as the dynamic EMG trials and the maximal amplitude of the three trials was used for EMG normalization. The EMG data for each throw were normalized to the maximal amplitude obtained during MVC for each of the respective muscles: pectoralis major, infraspinatus, serratus anterior, latissimus dorsi, upper, middle, and lower trapezius (normalized EMG hence forth called nEMG). The peak nEMG of each muscle in the periods before and after the cocking phase as well as at ball release were obtained.

### Statistical Analysis

The required sample size was estimated from a pilot study performed in our lab. The data distributions were tested for normality with the Shapiro–Wilk test. To assess the differences in joint kinematics between the two groups, an independent sample *t*-test (pain vs. no pain) was used. Means and standard deviations (SD) were calculated for all data. *P*-values of ≤ 0.05 were considered statistically significant.

To evaluate differences in EMG activity before and after maximal shoulder extension, a 2 (groups: pain, no pain) × 3 (events: pre-, post maximal shoulder extension and at ball release) × 7 (muscles) measures analysis of variance (ANOVA) was performed. If a difference in activity was found, two-way ANOVA per event was also performed. Timing was compared per event by a 2 (groups: pain, no pain) × 7 (muscles) ANOVA. Holm–Bonferroni post hoc tests were used to identify during which period potential differences in EMG activity and timing occurred. If assumption of the sphericity was violated, the Greenhouse–Geisser adjustments of the *p*-values were reported. All results are presented as means ± standard deviations. Effect sizes were evaluated with $\eta_{\text{p}}^{2}$ (partial eta squared), where 0.01–0.06 constitutes a small effect, 0.06–0.14 a medium effect, and >0.14 a large effect (Cohen, 1988). Statistics were analyzed in SPSS version 27.0 (IBM Corp., Armonk, New York, USA).

## Results

The two groups were comparable on anthropometric parameters (age, height, BMI) except for mass and practice time for strength and cardio training (Table 1).

**Table 1:** Anthropometry and training experience per group.

	**Players with Pain** **(*n* = 15)**	**Controls, players without pain** **(*n* = 15)**	***P*-value**
Age (years)	22.2 ± 2.9	20.4 ± 2.6	0.12
Height (m)	1.76 ± 0.07	1.72 ± 0.05	0.12
Mass	73.8 ± 9.7	66.9 ± 3.9	0.05^*^
BMI (kg/m^2^)	23.7 ± 3.1	22.3 ± 1.2	0.18
Years of playing handball	13.8 ± 3.1	13.7 ± 3.1	0.68
Years of playing professional handball	2 ± 2.4	2.4 ± 2.7	0.71
Practice per week of handball (Hrs.)	6.2 ± 1.1	6.5 ± 1	0.55
Practice per week of strength training (Hrs.)	2.5 ± 0.7	4.2 ± 1.7	0.004^*^
Practice per week of cardio training (Hrs.)	1.5 ± 0.8	2.3 ± 0.9	0.05^*^
Practice per week of prophylactic (Hrs.)	1.1 (0.7)	1.3 (1.3)	0.70

* *Indicates a significant difference between the two groups where p ≤ 0.05*.

### Throwing Kinematics

**Figure 4:**
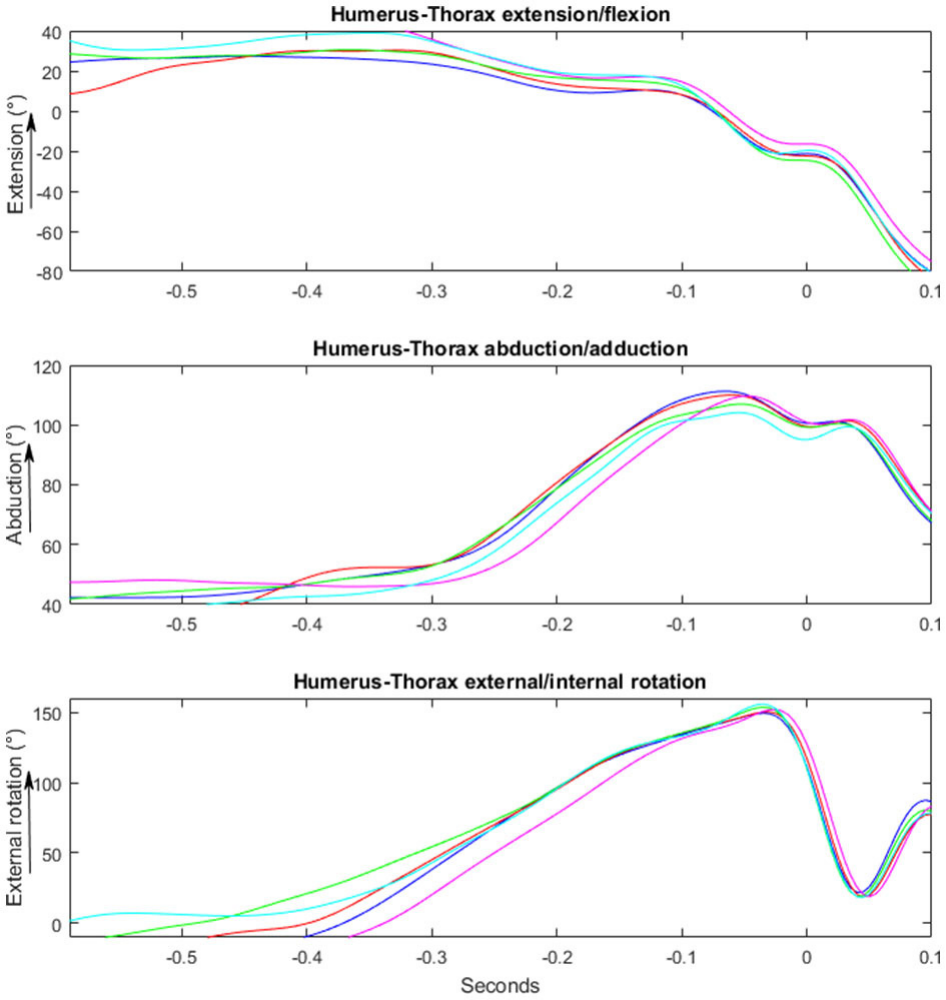
Illustrating a kinematic movement of a standing overhead throw performed by a player playing with no pain. Y-axis shows the changes in degrees/°. X-axis shows the time in milliseconds, where 0 is the time of ball-release.

**Figure 5:**
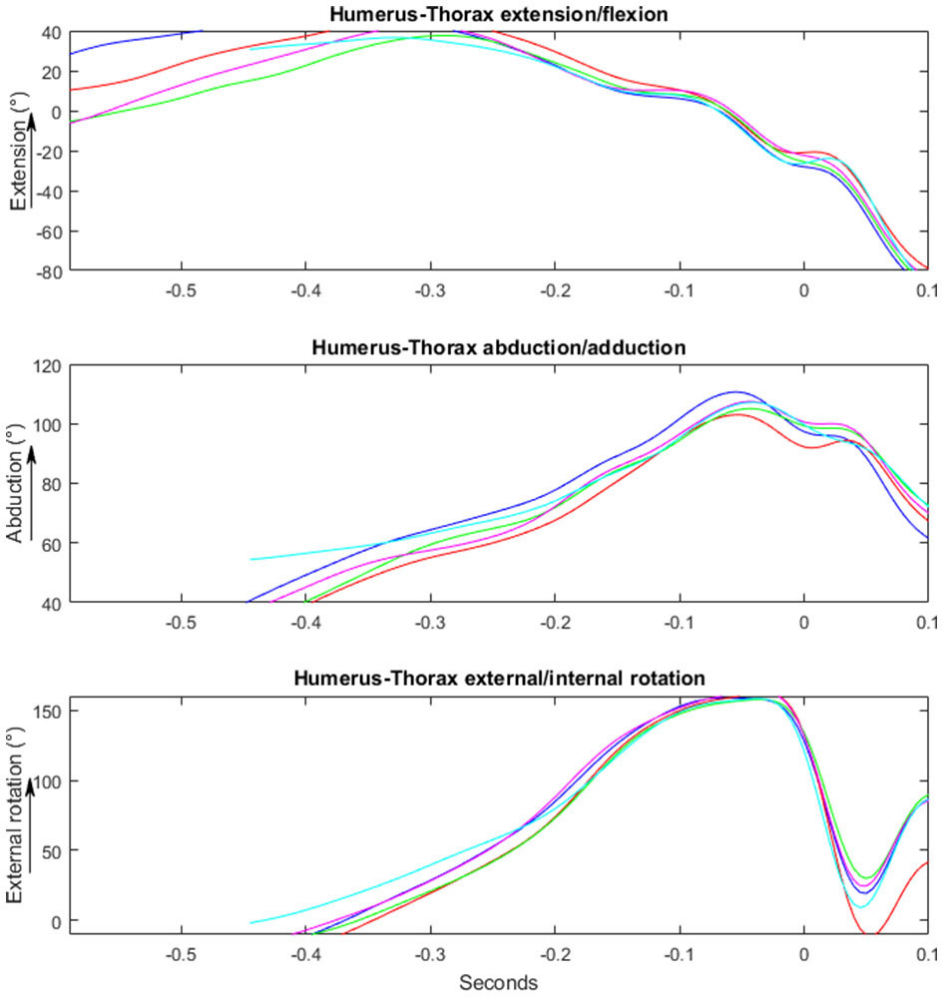
Illustrating a kinematic movement of a standing overhead throw performed by a player playing with pain. Y-axis shows the changes in degrees/°. X-axis shows the time in milliseconds, where 0 is the time of ball-release.

**Figure 6:**
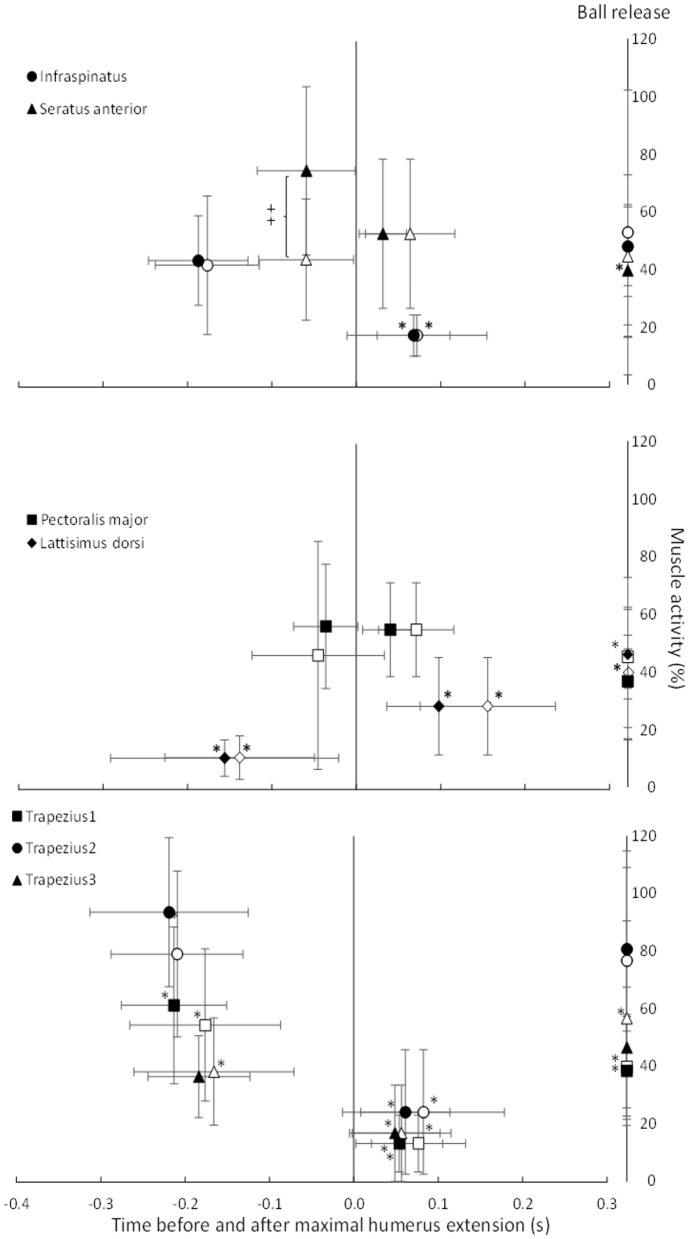
Average (±SD) peak EMG activity and timing per muscle before and after maximal shoulder extension (0.0) and at ball release per group (pain group: open symbols, no pain group: closed symbols). *Indicates a significant difference in peak EMG activity for this muscle for this group at this event compared with the two other events. ^‡^Indicates a significant different between the groups for this peak EMG activity on a *p* ≤ 0.05 level.

Kinematic analyse of the entire throwing movement in the two groups are presented in Figures 4, 5. No significant differences in maximal angles and angles at ball release were found between the two groups [*F*_(1, 29)_ ≤ 3.2, *p* ≥ 0.081; $\eta_{\text{p}}^{2}$ ≤ 0.11]. However, the occurrence of the maximal shoulder flexion [*F*_(1, 29)_ = 6.4, *p* = 0.019; $\eta_{\text{p}}^{2}$ = 0.22) and external rotation angles [*F*_(1, 29)_ = 6.9, *p* = 0.014; $\eta_{\text{p}}^{2}$ = 0.21] were closer to ball release in the no pain group than in the pain group (Table 2). No significant differences in maximal angular joint velocities [*F*_(1, 29)_ ≤ 1.1, *p* ≥ 0.29; $\eta_{\text{p}}^{2}$ ≤ 0.04] and their time of occurrence [F_(1, 29)_ ≤ 1.9, *p* ≥ 0.175; $\eta_{\text{p}}^{2}$ ≤ 0.07) were found for any of the joint movements (Figure 6).

**Table 2:** Maximal shoulder angles (°)±SD and timing(s) before ball release and joint angles at ball release.

**Parameter**	**Pain**		**No pain**		**At ball release**	
	**Maximal**	**Timing**	**Maximal**	**Timing**	**Pain**	**No pain**
**Shoulder joint**
Extension	25.8 ± 12.6	0.251 ± 0.081	28.7 ± 13.3	0.183 ± 0.056^*^	−19.2 ± 9	−12.6 ± 10.4
Abduction					89.3 ± 11.3	86.0 ± 10.7
External rotation	158.6 ± 10.6	0.036 ± 0.010	156.5 ± 13.1	0.029 ± 0.007^*^	131.5 ± 16.8	125.3 ± 13.6
Internal rotation	22.3 ± 13		21.8 ± 14.5			
**Trunk**
Trunk rotation	−98.3 ± 9.6		−97.8 ± 11.5		19.6 ± 8.6	18.5 ± 9.1
Pelvis rotation	−79.7 ± 8.2		−80.3 ± 12.8		18.8 ± 7.5	17 ± 10.1

* *Indicates a significant difference between the pain and the no pain group where p ≤ 0.05*.

### Peak EMG Activity

**Figure 7:**
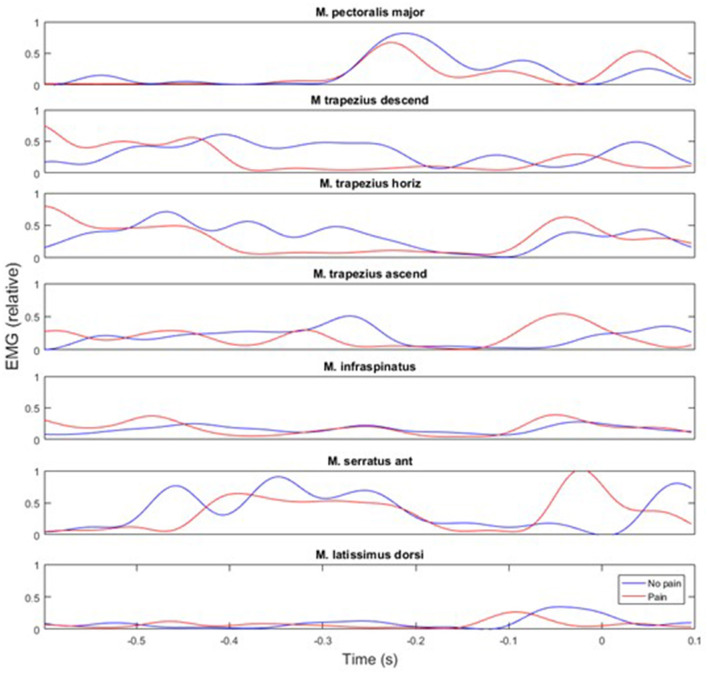
Representative example of muscle activity during a throw performed by an athlete with shoulder pain (red line) and no pain (blue line). Point 0 is defined as ball release.

**Figure 8:**
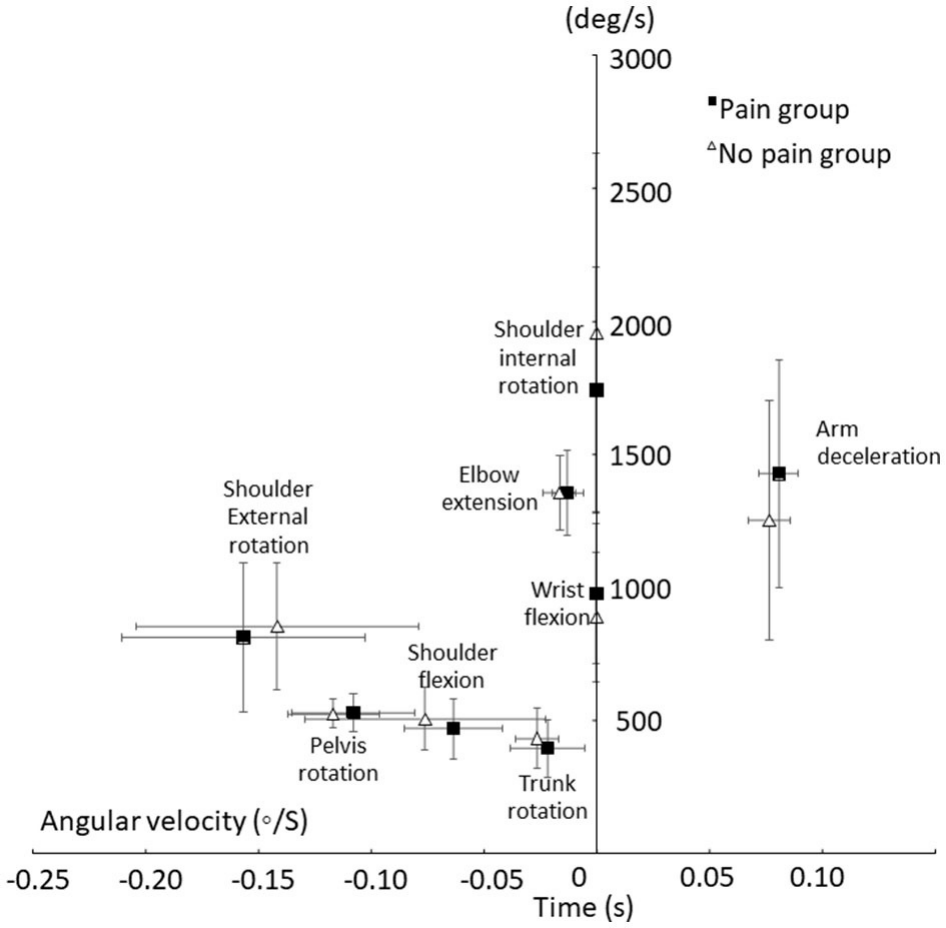
Average (±SD) peak shoulder angular velocities and arm angular deceleration velocity and time of occurrence until ball release for the pain and no pain group.

During an overhead throw, the development of muscle activity changed through the movement. Peak activity appeared differently between an athlete playing with and without shoulder pain (Figure 7). The peak nEMG was measured before and after the cocking phase and at ball release during the standing throw and was significantly different between muscles [*F*_(3.15, 63.16)_ = 14.5, *p* < 0.001; $\eta_{\text{p}}^{2}$ = 0.42], events [*F*_(1.37, 27.57)_ = 19.5, *p* < 0 .001; $\eta_{\text{p}}^{2}$ = 0.49] and event^*^muscle interaction [*F*_(5.27, 105.38)_ = 32.7, *p* < 0.001; $\eta_{\text{p}}^{2}$ = 0.62]. No significant group [*F*_(1, 23)_ = 0.43, *p* = 0.521; $\eta_{\text{p}}^{2}$ = 0.02] or group^*^event interaction [*F*_(1.38, 27.57)_ ≤ 1.6, *p* ≥ 0.089; $\eta_{\text{p}}^{2}$ ≤ 0.07] effects were found. However, when evaluated per event, the group effect approached the level of significance [*F*_(1, 23)_ = 4.2, *p* = 0.053; $\eta_{\text{p}}^{2}$ = 0.15). *Post-hoc* comparison showed that the different parts of the trapezius all had their highest activity before the maximal humeral extension, had the lowest activity during acceleration, and during ball release it approached the same level of activity as in the cocking phase. The latissimus dorsi had the lowest activity during the cocking phase and increased to the highest activity at ball release (Figure 7). The infraspinatus had similar activation during the cocking phase and at ball release, while activation decreased during the acceleration phase. The pectoralis muscle did not change peak activity significantly in the different phases, while the serratus anterior was the only muscle that showed a significant difference in activity between groups. During the cocking phase, the no pain group had significantly higher peak activity than the pain group, which decreased to a significantly lower peak activity at ball release, while the pain group had similar peak EMG activity during all three events (Figure 8). Furthermore, during the cocking phase, the middle trapezius had the highest peak activity followed by the serratus anterior, upper trapezius, pectoralis major, lower trapezius and infraspinatus. The latissimus dorsi had significantly lower peak activity than all other muscles during this phase, while during the acceleration phase the highest peak activities were found in the pectoralis (57%) and serratus anterior (55%) muscles. All other muscles had a lower but similar peak activity during this phase of 17–28% of maximal MVC. At ball release only the lower trapezius had significantly higher peak EMG activity (83%) than the rest of the muscles (Figure 8).

### Timing of Peak Muscle Activity

A significant effect in timing between the muscles was found [*F*_(3.79, 83.27)_ ≥ 7.8, *p* ≤ 0.001; $\eta_{\text{p}}^{2}$ ≥ 0.26] during both phases, while no significant group [*F*_(1, 22)_ ≤ 2.6, *p* ≥ 0.119; $\eta_{\text{p}}^{2}$ ≤ 0.11] or interaction [*F*_(3.79, 83.27)_ ≤ 1.1, *p* ≥ 0.37; $\eta_{\text{p}}^{2}$ ≤ 0.05] effects were found. *Post-hoc* comparison revealed that during the cocking phase, the peak activity of the trapezius appeared earliest, together with the infraspinatus and latissimus dorsi. The pectoralis major and serratus anterior reached peak activity significantly closer before maximal humeral extension than the other muscles. During the acceleration phase, only the latissimus dorsi peak activity was reached closer to ball release than all other muscles (Figure 8).

## Discussion

The main findings of this explorative study were that the maximal joint angles and angular velocities did not differ between the groups. However, group differences were observed in the earlier time of occurrence and time spent in maximal shoulder extension and external shoulder rotation in the pain group compared to the no pain group. The pain group also showed a decreased peak muscle activity in the serratus anterior during the cocking phase compared to the no pain group.

The only differences in kinematics between the two groups were the earlier time of occurrence of the maximal shoulder extension and external shoulder rotation, in which the pain group reached these peak joint angles earlier before ball release. The earlier occurrence of the maximal shoulder extension could be a mechanism of adaptation to avoid pain, because this joint angle indicates the transition from the cocking phase to the ball acceleration phase. A longer ball acceleration phase may allow lower peak acceleration around the different joints, and thus, lower force-induced stress to passive joint tissues, without compromising throwing velocity. In other words, this may be a way of reducing the pain-causing stresses in the anterior and inferior parts of the shoulder joint capsule, as well as to the medial collateral ligament of the elbow. Furthermore, greater shoulder flexion (19°) at ball release was seen in the pain group compared the no pain group (12°), which was marked by a non-significant (*p* = 0.081), yet medium effect size (0.11). The different position of shoulder flexion at ball release may be an adaption to avoid pain and create less stress on the glenohumeral joint. This position may influence the time the players have in the deceleration phase and follow-through. That would require a larger eccentric muscle contraction of the posteriorly placed muscles in the pain group compared to the no pain group. However, kinetic calculations must be conducted to confirm this, which was not possible in the present study.

The only significant difference in muscle activity was found for the serratus anterior. The no pain group had higher peak activity during the cocking phase, which decreased to a significantly lower peak activity at ball release, while the pain group showed similar peak EMG activity during all three events (Figure 6). That higher activation of the serratus anterior during the cocking phase in the no pain group could be due to a potentially higher shoulder extension velocity (not measured) to move the ball up, backwards (cocking phase), increase the upward rotation of the scapula and create a potentially larger subacromial space. When moving the ball fast backwards, the eccentric activity of the serratus anterior must increase to stop the scapula retraction. In the pain group, this movement could be slower, as indicated with the earlier occurrence of maximal shoulder extension. A longer period of time in the end range may influence how effective the muscles are able to peak and optimize the kinematic chain and scapula-humeral movement. Furthermore, a non-optimal muscular activation of the scapular-humeral muscles is already identified as a risk factor in the literature due to the scapula's position of controlling the scapula-humeral rhythm and its position in transferring muscle power from the trunk to the humerus (Kibler et al., 1996, 2013a,b; Laudner et al., 2006, 2013; Myers et al., 2013; Pluim, 2013; Struyf et al., 2014). The observed differences in muscle activation pattern may also be a result of player specialization, i.e., when throwing, a pivot or wing player need to position their arm in a different position due to the presence of a defender or goalkeeper. The data were collected without a defender present, but the differences may also be an expression of a throwing technique adapted to the playing position, style and the anthropometric differences.

Only few differences in kinematics and muscle activation patterns were observed between the two groups. A significant group difference was found in the time spend doing strength and cardiovascular training. Although players were recruited from the same leagues/teams, big financial differences are known to exist between teams. The questionnaire did not cover financial position as a professional athlete. However, fulltime professional players have more time to train during the day and better conditions for optimizing the restitution period. Another consideration is if the extra amount of strength training has a prophylactic effect on the development of shoulder pain in the group and by that provides greater resistance to the acute and chronological training loads a team handball player experience during a match season. A study by Moller et al. showed how scapula dyskinesia and decreased strength in external rotators combined with an increased acute training load were risk factors for the development of shoulder pain (Moller et al., 2017). A lack of differences may also be a result of the variation of throwing styles between athletes. There are many ways to throw a ball fast, and each elite handball player has adapted their throwing technique to their balances in muscle strength and to the specific requirements of their sport. When an athlete experiences pain, she will thus adapt her movements and muscle activity pattern to avoid that pain (Laudner et al., 2006, 2013). Because the pain group consisted of athletes with different types of shoulder pain, these adaptations varied, so different solutions occurred in the changed motor patterns, reflecting very few differences between the pain and no pain group in kinematics and muscle activity. Thus, in future studies, the pain group could be categorized to investigate whether different types of shoulder pain and pathologies will result in different adaptations.

To our knowledge, this is the first paper to explore throwing kinematics and muscle activation in elite team handball players playing with or without pain. Due to the study design, we cannot conclude whether the observed group differences are a result of pain or a cause of pain. However, information regarding kinematic performance and muscle activation pattern could potentially serve to disclose non-optimal factors that may be addressed to optimize functional performance or in prevention programs before pain occur. In a clinical perspective, information of decreased or increased muscle activity in the functional movement, may be used to adjust rehabilitations programs, but also to recreate “normality” within an athlete's own biomechanical movement pattern before and after an injury appear.

### Limitations

This study is novel as it is the first attempt to explore potentially differences in throwing kinematics and muscle activation in team handball players playing with and without shoulder pain. However, limitations do exist. Besides the possible variation in individual throwing techniques discussed above, other limitations related to technology and laboratory settings may increase the variation or reduce the chance of detecting differences. The study was performed in a controlled environment, where the laboratory setting may limit or change the players throwing performance due to lack of goalie, defender, tackles or experience in a test situation. Also, in order to minimize EMG crosstalk, international recommendations for sensor placement procedures were followed. However, data collected in this study were obtained in a functional explosive movement, and it is possible crosstalk may have influenced the results. Furthermore, the analysis of muscle activation patterns offers challenges as variation in the timing of peak amplitudes may be influenced by the presence of more than one peak in each phase of the throw in some subjects. The choice of a lowpass filter with low cut-off would reduce the influence of this problem, but individual coordination patterns like this cannot be ruled out, and this may possibly have contributed to an increased variation. Also, the amplitude of the selected peaks may be influenced by inter-subject variations which in-turn may increase the variation of the normalized activation values. However, as all subjects were elite athletes used to perform maximal muscle contractions, it may be a less likely cause of error.

Nevertheless, access to similar research has been few and limited our possibilities to confirm or compare our results. Furthermore, the required sample size was based on a pilot study prior to the data collection. Future studies should include more participants or participants with a specific type of shoulder pain to decrease variance within groups and to detect possible between group differences easier.

## Conclusion

The purpose of the present explorative study was to investigate the throwing kinematics and muscle activation of a group female elite handball players playing handball with and without shoulder pain. The kinematic findings revealed that maximal shoulder extension and maximal external shoulder rotation occurred earlier within the pain group. Ultimately, the results of muscle activity showed that the no pain group had a significantly higher peak activity of the serratus anterior before the cocking phase compared to the pain group. No differences between groups were found in angular velocities and angular accelerations. The results indicate that handball players playing with pain differ in the timing of throwing kinematics, maximal humerus extension and external rotation, which could be the result of adaptive mechanisms to avoid pain. Players with shoulder pain had lower activity in the serratus anterior during the throw. However, further investigations are needed to establish if a relationship between overhead throwing kinematics and shoulder pain do exist, and if other types of throws influence handball players with pain the same way as a standing throw and whether fatigue in the shoulder affects the groups of handball players differently, which may influence prevention strategies against shoulder pain.

## Data Availability Statement

The raw data supporting the conclusions of this article will be made available by the authors, without undue reservation.

## Ethics Statement

The studies involving human participants were reviewed and approved by Danish Ethics Committee Denmark. The patients/participants provided their written informed consent to participate in this study.

## Author Contributions

JB, MKZ, and TPT: conceptualization. JB, TPT, BJ-K, MD, MKZ, and RT: methodology. JB and RT: software and supervision. TPT: data collection, writing, and original draft preparation. TPT, BJ-K, MKZ, RT, and JB: writing, review and editing. JB and BJ-K: project administration. All authors have read and agreed to the published version of the manuscript. All authors contributed to the article and approved the submitted version.

## Conflict of Interest

The authors declare that the research was conducted in the absence of any commercial or financial relationships that could be construed as a potential conflict of interest.

## Publisher's Note

All claims expressed in this article are solely those of the authors and do not necessarily represent those of their affiliated organizations, or those of the publisher, the editors and the reviewers. Any product that may be evaluated in this article, or claim that may be made by its manufacturer, is not guaranteed or endorsed by the publisher.
